# Moderate hypothermia within 6 h of birth plus inhaled xenon versus moderate hypothermia alone after birth asphyxia (TOBY-Xe): a proof-of-concept, open-label, randomised controlled trial

**DOI:** 10.1016/S1474-4422(15)00347-6

**Published:** 2016-02

**Authors:** Denis Azzopardi, Nicola J Robertson, Alan Bainbridge, Ernest Cady, Geoffrey Charles-Edwards, Aniko Deierl, Gianlorenzo Fagiolo, Nicholas P Franks, James Griffiths, Joseph Hajnal, Edmund Juszczak, Basil Kapetanakis, Louise Linsell, Mervyn Maze, Omar Omar, Brenda Strohm, Nora Tusor, A David Edwards

**Affiliations:** a Centre for the Developing Brain, Division of Imaging Sciences and Bioengineering, King's College London, London, UK; b EGA Institute for Women's Health, University College London, London, UK; c Faculty of Engineering Science, University College London, London, UK; d UCL Hospitals NHS Foundation Trust, London, UK; e Department of Medical Physics, Guy's & St Thomas' NHS Foundation Trust, London, UK; f Division of Neonatology, Imperial College Healthcare NHS Trust, London, UK; g Faculty of Natural Sciences, Department of Life Sciences, Imperial College London, London, UK; h National Perinatal Epidemiology Unit, Nuffield Department of Population Health, University of Oxford, Oxford, UK; i Anesthesia and Perioperative Care, University of California San Francisco School of Medicine, San Francisco, CA, USA

## Abstract

**Background:**

Moderate cooling after birth asphyxia is associated with substantial reductions in death and disability, but additional therapies might provide further benefit. We assessed whether the addition of xenon gas, a promising novel therapy, after the initiation of hypothermia for birth asphyxia would result in further improvement.

**Methods:**

Total Body hypothermia plus Xenon (TOBY-Xe) was a proof-of-concept, randomised, open-label, parallel-group trial done at four intensive-care neonatal units in the UK. Eligible infants were 36–43 weeks of gestational age, had signs of moderate to severe encephalopathy and moderately or severely abnormal background activity for at least 30 min or seizures as shown by amplitude-integrated EEG (aEEG), and had one of the following: Apgar score of 5 or less 10 min after birth, continued need for resuscitation 10 min after birth, or acidosis within 1 h of birth. Participants were allocated in a 1:1 ratio by use of a secure web-based computer-generated randomisation sequence within 12 h of birth to cooling to a rectal temperature of 33·5°C for 72 h (standard treatment) or to cooling in combination with 30% inhaled xenon for 24 h started immediately after randomisation. The primary outcomes were reduction in lactate to N-acetyl aspartate ratio in the thalamus and in preserved fractional anisotropy in the posterior limb of the internal capsule, measured with magnetic resonance spectroscopy and MRI, respectively, within 15 days of birth. The investigator assessing these outcomes was masked to allocation. Analysis was by intention to treat. This trial is registered with ClinicalTrials.gov, number NCT00934700, and with ISRCTN, as ISRCTN08886155.

**Findings:**

The study was done from Jan 31, 2012, to Sept 30, 2014. We enrolled 92 infants, 46 of whom were randomly assigned to cooling only and 46 to xenon plus cooling. 37 infants in the cooling only group and 41 in the cooling plus xenon group underwent magnetic resonance assessments and were included in the analysis of the primary outcomes. We noted no significant differences in lactate to N-acetyl aspartate ratio in the thalamus (geometric mean ratio 1·09, 95% CI 0·90 to 1·32) or fractional anisotropy (mean difference −0·01, 95% CI −0·03 to 0·02) in the posterior limb of the internal capsule between the two groups. Nine infants died in the cooling group and 11 in the xenon group. Two adverse events were reported in the xenon group: subcutaneous fat necrosis and transient desaturation during the MRI. No serious adverse events were recorded.

**Interpretation:**

Administration of xenon within the delayed timeframe used in this trial is feasible and apparently safe, but is unlikely to enhance the neuroprotective effect of cooling after birth asphyxia.

**Funding:**

UK Medical Research Council.

## Introduction

Treatment of neonatal encephalopathy with moderate hypothermia is now standard care in several countries.^1^ Cooling to 33–34°C after birth asphyxia increases survival without impairments in childhood by about 15%, but roughly 25% of treated infants with moderate or severe encephalopathy die and 20% of survivors develop sensorimotor and cognitive impairments.

Additional therapies might further improve outcomes in these infants. Several neuroprotectants are effective in experimental studies; the challenge is to find the most promising candidate treatment to take forward to clinical trials. In a comparative review of potential neuroprotectants,^2^ inhaled xenon was rated highly but, because of the need for specialist equipment and training, there were concerns about cost and ease of administration.

Xenon is a monoatomic gas that rapidly crosses the blood–brain barrier. It is an approved inhalational anaesthetic at a minimum alveolar concentration of 60–70% in adults and is not associated with adverse cardiovascular effects, or anaesthetic-associated neurotoxic effects.^3^ Broad interest in xenon as a potential neuroprotectant is based on strong experimental evidence, but the drug is difficult to use in clinical practice. Xenon might provide neuroprotection after asphyxia by different mechanisms. It is an inhibitor of NMDA glutamate receptors and so could reduce neuronal injury caused by excessive glutamate concentrations and lessen seizures, and it reduces apoptosis by activation of anti-apoptotic factors.^4^, ^5^, ^6^ Xenon reduced cerebral injury in models of hypoxic ischaemic injury in different animal species and the neuroprotective effect was stronger when xenon was used in combination with cooling.^7^ The combination of 20% xenon with cooling to 35°C provided synergistic neuroprotection in both in-vitro and in-vivo models, with improvement in function for 30 days, but neither intervention alone was effective.^8^

**Research in context**

**Evidence before this study**

We searched PubMed with the terms “xenon neuroprotection” and “xenon hypothermia” for all articles published in English until July 23, 2015. Our search returned several preclinical studies, in which the neuroprotective effects of xenon after asphyxia were shown; these effects were enhanced when xenon was used in combination with hypothermia. Investigators of clinical studies had reported the feasibility of treatment with xenon in combination with hypothermia for neuroprotection in neonates after birth asphyxia and in adults after cardiac arrest, but we found no reports of neuroprotective effects associated with this therapy.

**Added value of this study**

Our trial is the first randomised clinical study of the neuroprotective effects of xenon in combination with hypothermia after birth asphyxia. The treatment regimen that we used is generally applicable in high-resource settings, and we assessed it with qualified cerebral magnetic resonance endpoints. Our proof-of-concept study showed that, in the complex situation of neonatal care, delayed intervention with xenon beyond 6 h after birth does not have additional neuroprotective effects compared with induction of hypothermia alone after birth asphyxia.

**Implications of all available evidence**

Strong experimental evidence supports the use of xenon as a neuroprotectant, but treatment with 30% xenon for 24 h begun more than 6 h after birth combined with early hypothermia is unlikely to improve clinical outcomes compared with hypothermia alone after birth asphyxia. Qualified magnetic resonance biomarkers offer the potential to speed up the assessment of promising neuroprotective treatments before a large pragmatic trial, would substantially reduce opportunity costs, and could lead to redirection of future research.

In adults with stroke, many neuroprotectants that were effective in preclinical studies were not associated with any benefits in large randomised trials.^9^ In view of the neuroprotective effect of cooling after birth asphyxia, a study of about 750 infants would be needed to detect a further 10% improvement in neurological outcomes with additional or modified therapy.^10^ The substantial financial and opportunity costs associated with large pragmatic clinical trials that yield negative results can be avoided by first assessing candidate treatments in small proof-of-concept trials, in which qualified biomarkers and surrogate endpoints are used to test efficacy in the clinical context. Treatments that show promise at this stage are candidates for large definitive trials with clinical endpoints.

In neonates after hypoxic ischaemic injury, the ratio of cerebral lactate to N-acetyl aspartate (assessed with magnetic resonance spectroscopy [MRS]) and fractional anisotropy (FA), a measure of tissue integrity in white matter tracts measured by diffusion tensor MRI, have been used in work in animals to assess potential neuroprotectants and can be used to predict subsequent neurological outcomes after birth asphyxia, including in infants treated with moderate hypothermia.^11^, ^12^, ^13^, ^14^

To assess whether the combination of cooling with inhaled xenon—administered at a concentration and within a timeframe suitable for general clinical application—could further improve neurological outcomes after birth asphyxia and neonatal encephalopathy, we compared the effects of combined therapy with cooling alone on the lactate to N-acetyl aspartate ratio in the thalamus and FA in white matter tracts within 15 days of birth.

## Methods

### Study design and participants

Total Body hypothermia plus Xenon (TOBY-Xe) was a proof-of-concept, pragmatic, open-label, parallel-group randomised controlled trial at four UK neonatal intensive-care units in London (University College Hospital, St Thomas' Hospital, Queen Charlotte and Chelsea Hospital) and Liverpool (Liverpool Women's Hospital). The National Perinatal Epidemiology Unit (University of Oxford, Oxford, UK), was the coordinating centre for the trial, and managed the study.

Infants were eligible if their gestational age was 36–43 weeks and they had at least one of the following: Apgar score of 5 or less 10 min after birth; continued need for resuscitation, including endotracheal or mask ventilation, 10 min after birth; or acidosis (defined as pH <7 or base deficit >15 mmol/L, or both, in umbilical cord blood or any blood sample) within 1 h of birth. Furthermore, eligible infants showed signs of moderate to severe encephalopathy, consisting of altered state of consciousness (reduced or absent response to stimulation), hypotonia or severe hypotonia, and abnormal primitive reflexes (weak or absent suck or Moro response), and had moderately or severely abnormal background activity for at least 30 min or seizures as shown by amplitude-integrated EEG (aEEG).^15^

We excluded infants if cooling was started after age 6 h or if they were older than 12 h at randomisation. We also excluded infants with an oxygen requirement of greater than 60%, those who needed nitric oxide inhalation or ventilation with a high-frequency oscillator, those who needed extracorporeal membrane oxygen, and those with major congenital abnormalities.

Infants were recruited and assessed on admission by study personnel and cared for in participating centres. Infants born at hospitals that refer patients to the participating neonatal intensive-care units were also eligible for inclusion. Transport teams provided written information about the study for the parents of infants from referring hospitals, and study personnel assessed the infants on admission to the participating centre.

The trial was approved by the UK National Research Ethics Service (approval number 10/H0707/33). Parents provided written informed consent; if neither parent was available, consent was first obtained by telephone and then written consent was obtained at the earliest opportunity. Consent was reaffirmed within 24 h of receiving written consent.

### Randomisation and masking

Eligible infants were randomly assigned (1:1) to cooling plus inhaled xenon or cooling only. Assignment to a treatment group was overseen by the National Perinatal Epidemiology Unit, and was done through a secure web-based system with a computer-generated randomisation sequence, with telephone back-up. Minimisation was used to ensure balance of treatment assignment among infants with moderate or severe grades of abnormality on aEEG and within each participating centre. Masking of investigators and parents to allocation was not practical because of the need for a special ventilator to administer xenon, and thus the trial was open label. However, investigators who assessed the primary outcome measures—ie, NT who assessed MRI data and AB who assessed MRS data—were masked to treatment allocation.

### Procedures

We used servo-controlled equipment to cool all infants to a target rectal temperature of 33·5°C for 72 h starting within 6 h of birth. If cooling equipment was not available at the referring hospital, passive cooling was commenced and active cooling was started by the transport team and continued during transport to the treatment centre. Infants in the inhaled xenon group also received 30% xenon (Lenoxe, Air Liquide, Paris, France) through an uncuffed endotracheal tube connected to a recirculating device developed for the trial (SLE, Croydon, UK).^16^ The system provided automated control of xenon, air, and oxygen mixture and continuous monitoring of xenon, oxygen, and carbon dioxide concentrations in inhaled gas. Xenon was commenced immediately after randomisation and continued for 24 h. After xenon administration ended, the infant was ventilated with a standard ventilator according to the unit's practice.

All MRS and MRI studies were done with 3·0 Tesla systems (Philips Healthcare, Best, Netherlands) at each centre. The trial research physicist (GF) undertook rigorous standardisation and a quality-control programme with phantoms and repeated scanning. Comparability test objects were transported from site to site during the project. An adult volunteer was imaged at each site periodically to provide direct comparison data.

Images were obtained according to a standard protocol that included T1-weighted and T2-weighted diffusion tensor MRI with 32 non-collinear directions, MRS from a single voxel on the left thalamus, and motion tolerant T1 and T2 structural scans. We used a study-specific SmartExam card (Philips Healthcare) to aid the planning of the various sequences (appendix). Total examination time for the study protocol was around 1 h. The SENSE 8 channel head coil (Philips Healthcare) was used for all infants.

### Outcomes

The primary outcomes were reduction in lactate to N-acetyl aspartate ratio in the thalamus on MRS or preserved FA in the posterior limb of the internal capsule on diffusion tensor MRI, as assessed by tract-based spatial statistics, an automated observer-independent method of aligning FA images from several patients to allow group-wise comparisons of diffusion tensor imaging data free from partial volume effects.^17^, ^18^

Secondary outcomes assessed before discharge from hospital were maximum Thompson hypoxic ischaemic encephalopathy score (range 0–22, with higher scores corresponding to worse encephalopathy);^19^ neurological examination at discharge from treatment centre (which were done by experienced nominated physicians at each treatment centre);^20^ occurrence of seizures; intracranial haemorrhage; persistent hypotension; pulmonary haemorrhage; pulmonary hypertension; prolonged blood coagulation time (activated partial thromboplastin time >41 s or international normalised ratio >3); thrombocytopenia (platelet count <150 × 10^9^ per L); major venous thrombosis; cardiac arrhythmia (heart rate <80 beats per min); culture-proven late-onset sepsis; necrotising enterocolitis; pneumonia; pulmonary air leak; anuria or urine output of less than 0·5 mL/kg/h for 24 h; age at which full oral feeding was achieved; and duration of hospital stay. We also measured the grade of abnormalities on visual analysis of MRI (scored 0–11, with higher scores corresponding to worse abnormalities).^21^ We did not compare aEEG results between groups after randomisation as initially planned, because during the study we noted that xenon treatment suppressed the reading, hindering a comparative analysis.^6^

Non-serious adverse events and reactions were reported on the data collection forms, but adverse events commonly associated with neonatal encephalopathy were not recorded. Serious adverse events that we monitored were deaths, hypertension (mean blood pressure >85 mmHg), hypotension (mean blood pressure <25 mmHg), cardiac arrhythmia (severe bradycardia [heart rate <60 beats per min] or ventricular arrhythmia), and inability to achieve adequate ventilation despite appropriate adjustment of ventilator settings.

### Statistical analysis

The National Perinatal Epidemiology Unit had data entry and management functions, provided an OpenClinica clinical database system, and did most of the analyses (it was masked to treatment allocation). Sample size was estimated primarily for detection of differences in the geometric mean lactate to N-acetyl aspartate ratio between groups because this value was greater than the number needed to detect a difference in FA. Based on assumptions from previous data, and allowing for a mortality rate of 20% before 15 days, a study of 138 infants would have 80% power to detect a geometric mean ratio of lactate to N-acetyl aspartate of 0·6, with a coefficient of variation of 1·2.^14^ A geometric mean ratio of 1 suggests no difference in mean values between the two groups, whereas a ratio of less than 1 favours the intervention group.

For changes in FA detected with tract-based spatial statistics, power was estimated by computational modelling and previous data, which suggested that with 80% power and a two-sided 5% significance level, a 10% change in FA would be detected in a study of 60 infants (higher FA shows less tissue damage).^11^, ^22^ Analysis of changes in FA induced by neuroprotective hypothermia showed that a substantial clinical effect was associated with changes of 10–20%.^11^, ^14^

All infants for whom magnetic resonance data were available were analysed in the groups that they were randomly allocated to, irrespective of allocation or protocol deviation. Diffusion tensor MRI and MRS analyses were done masked to treatment allocation (data were anonymised and allocation group was not included). We could not adjust for the minimisation factors used during randomisation because of small numbers.

We used linear regression to analyse differences between the intervention and control groups in mean thalamic ln(lactate/N-acetyl aspartate). The difference between the natural logarithm of two ratios is equivalent to the ratio of geometric means—ie, the geometric mean ratio. A ln(x + 1) transformation was used because of the presence of zero values for the lactate to N-acetyl aspartate ratio in the data. We used Randomise (a program for non-parametric permutation inference for neuroimaging data; v2.9) to analyse data for FA, controlling for postmenstrual age, with the two-sample *t* test with nuisance variable options.^23^

We did two sets of analysis for the two primary outcomes: the first included all infants who had MRI, and the second excluded those who subsequently died before discharge to account for a possible differential rate of scanning among these cases. We prespecified a subgroup analysis of lactate to N-acetyl aspartate by severity of abnormality of aEEG at randomisation. We also explored the effect of the time from birth to start of xenon therapy on the lactate to N-acetyl aspartate ratio and on FA, and the relation between these measures and neurological findings at discharge. A p value of 0·05 (two-sided 5% significance level) was deemed significant for the primary outcomes, and a p value of 0·01 (two-sided 1% significance level) was deemed significant for the exploratory analyses of secondary outcomes. We used Stata/SE (version 13.1) for all analyses.

A data monitoring committee oversaw the study, and the members of the committee had no involvement in the day-to-day running of the trial. Decisions and recommendations made by the data monitoring committee were communicated to the trial steering committee in writing. The data monitoring committee met four times between September, 2010, and December, 2013, and received reports of analyses of safety data after incremental enrolments of around 25 infants. A study statistician, who attended the open part of the meeting only, provided the reports. Although the charter allowed interim assessment of efficacy, no interim analyses of efficacy were done: they were not requested by the data monitoring committee because the study did not include the pre-specified sample size. The committee decided to look only at safety outcomes.

This trial is registered with ClinicalTrials.gov, number NCT00934700, and with ISRCTN, as ISRCTN08886155.

### Role of the funding source

The funder of the study had no role in study design; data collection, analysis, or interpretation; or writing of the report. The study statisticians (OO and LL) had full access to all the data in the study, and provided data to the corresponding author after data analysis was completed. Summary data were provided to all the authors. The corresponding author had final responsibility for the decision to submit for publication.

## Results

The study was done from Jan 31, 2012, to Sept 30, 2014. We screened 220 infants for eligibility, 92 of whom were enrolled up to completion of the enrolment period (figure 1). Failure to recruit the target sample size of 138 was primarily due to the closing of recruitment at the Liverpool participating centre after one infant had been enrolled because of incompatible configuration of scanner magnet gradient coils. 46 infants were randomly assigned to the cooling only group, and 46 to the xenon group (figure 1). Baseline clinical characteristics of the infants who were assigned to the two groups were broadly similar (table 1).

All infants allocated to the cooling plus xenon group received xenon. Ventilation with xenon commenced a median of 10·0 h (IQR 8·2–11·2, range 4·0–12·6) after birth and continued for a median of 24 h (IQR 24–24). The mean concentration of inhaled xenon was 32·2% (SD 6·9). Ventilation with xenon was started within 6 h of birth in seven (15%) of 46 infants and after 12 h in five (11%) infants (range 12·1–12·6 h), and was discontinued in two (4%) infants before 24 h because of increasing oxygen requirements due to persistent pulmonary hypertension. Median xenon leakage was 12 mL/min (IQR 10–15).

Cerebral magnetic resonance scans were done in 37 (80%) of 46 infants a mean of 5·8 days (SD 2·0) after birth in the cooling group and 41 (89%) of 46 infants at 6·0 days (2·1) after birth in the cooling plus xenon group. Lactate to N-acetyl aspartate ratio in the thalamus and FA values in the posterior limb of the internal capsule were similar in the two groups. The thalamic geometric mean ratio of lactate to N-acetyl aspartate was 1·09 (95% CI 0·90 to 1·32) and mean difference in FA was −0·01 (−0·03 to 0·02); exclusion of deaths from the analysis did not significantly affect results (table 2).

Two adverse events were reported during the study, both in the cooling plus xenon group. Subcutaneous fat necrosis, which is associated with cooling therapy, was noted in one, and transient desaturation during the MRI (done after cessation of cooling and xenon) in another. No serious adverse events occurred, but nine (20%) infants in the cooling group and 11 (24%) in the cooling plus xenon group died (relative risk 1·22, 99% CI 0·44–3·41). Neither event rates of adverse outcomes and other clinical measures examined before discharge from hospital (table 3) nor the distribution of MRI scores between groups (table 4) differed significantly.

Lactate to N-acetyl aspartate ratio results did not differ significantly according to severity of abnormality of the aEEG at randomisation (geometric mean ratio 1·02 [95% CI 0·97–1·09] in the moderately abnormal aEEG group *vs* 1·09 [0·87–1·36] in the severely abnormal aEEG group; p_interaction_=0·80). No significant relations were noted between time from birth to start of xenon therapy and the magnetic resonance measures (Spearman's correlation −0·14 for FA and −0·05 for the lactate to N-acetyl aspartate ratio). When we compared infants with normal or mildly abnormal results on neurological examination at discharge and those with moderately or severely abnormal results, the difference in means was 0·52 (95% CI 0·46–0·60; p<0·0001) for the lactate to N-acetyl aspartate ratio and 0·03 (0·01–0·06; p=0·02) for FA in the posterior limb of the internal capsule.

## Discussion

Our results showed that when xenon was used in a real-world context—ie, when it is given only at specialist centres and not during transport in a population of referred infants—qualified biomarkers of brain damage were not significantly affected and there was no treatment benefit. The use of magnetic resonance biomarkers to assess potential treatments rapidly and at low cost might be applicable to other neuroprotective therapies.

We planned to enrol 138 infants to acquire primary outcome data for 111 infants, but only had data for 78 infants for the lactate to N-acetyl aspartate ratio and for 73 infants for the FA analyses. The study was powered primarily for lactate to N-acetyl aspartate ratio, but we used data from a previous study of successful neuroprotection with hypothermia to predict a 10–20% increase in FA because of treatment, for which a study size of 60 infants would be sufficient to detect a clinically significant difference of 10%.^11^, ^14^ This estimate was supported by the results of an in-silico modelling study^22^ in which the effect of changing FA values on a tract-based spatial statistics study was simulated and the number of voxels showing a significant difference by FA change was estimated; the results showed that a study size of 60 infants would be sufficient to detect clinically important differences in FA between the study groups. Thus, our study was adequately powered to detect changes in FA.^22^ The model was validated with infant data, showing that the model predicts real-world data accurately. Our trial is underpowered for the lactate to N-acetyl aspartate ratio, but provides a reliable estimate of lack of biological effect through tract-based spatial statistics, and that all outcome measures are concordant is relevant.

The duration of the enrolment period was 32 months, 2 months longer than was planned in the protocol, and we did not seek to extend that period largely because the sample size needed to detect a significant change in FA had been reached, and our goal was a rapid analysis of the suitability of the intervention for a large pragmatic trial. Although the study size was smaller than initially planned, judging by our results there is only a remote possibility that outcomes would materially change if we had enrolled the planned number.

We initially planned to include three participating centres in the trial, but later sought a fourth centre to ensure that recruitment would be to target. However, during the quality-assurance check done after the first baby was recruited at the fourth centre, the MRI scanner gradient set-up was shown to differ from that of the other scanners resulting in a potential discrepancy of 10% in the data. We therefore closed recruitment at that centre, which reduced the number of infants who could be recruited. This issue shows the complexity of establishing magnetic resonance biomarkers across several sites.

Follow-up of the study cohort is ongoing according to our clinical practice and because of the novelty of the intervention. Xenon's lack of efficacy despite promising experimental studies in animals can be explained in several ways. The timing, dose, and duration of treatment with inhaled xenon might have been suboptimum. Our regimen was based on clinical and safety factors: higher doses had not previously been given for such prolonged periods and could not be delivered to infants with substantial oxygen requirements, and earlier intervention would not be feasible for most infants born outside treatment centres. In a feasibility study in newborn infants given up to 50% xenon for 3–18 h, xenon was begun a median of 11 h (range 5–18) after birth;^24^, ^25^ earlier treatment might be possible if xenon can be delivered during transport to a specialist centre, but even then substantial delays are probable.

We also based the treatment regimen on experimental studies,^7^, ^8^ the results of which suggested both that the therapeutic window for neural rescue was extended with cooling and that the combination of cooling with xenon has synergistic or at least additive neuroprotective efficacy and thus subanaesthetic doses could be effective. In addition to neuroprotective effects through inhibition of NMDA receptors, which have an important role in the early phase of reperfusion injury, xenon reduces apoptotic cell death, which occurs in the later phase of reperfusion injury. Thus, the hypothesis that delayed treatment with xenon in combination with early hypothermia might have neuroprotective effects is plausible. However, the effects of delayed treatment with xenon are variable in work in animals and no studies have been done of hypothermia augmented with xenon starting 6 h after insult.^26^, ^27^ We did not find a relation between timing of xenon inhalation in the time range used in this study and the magnetic resonance biomarkers. Since only seven (15%) of 46 infants in the xenon group received xenon by 6 h, we cannot exclude the possibility that starting xenon within 6 h of birth might be beneficial.

Experimental studies of cooling for neuroprotection suggest that treatment for 72 h is needed when initiation is delayed.^28^ Early clinical studies of MRS in neonates also showed that the secondary reperfusion phase of injury after asphyxia lasts about 72 h, so our 24 h treatment might have been too short.^29^ Further evidence that the optimum duration of treatment could be longer than 24 h was provided by our previously reported finding of a transient recurrence of seizures after discontinuation of xenon.^6^ However, much shorter treatment durations are neuroprotective in experimental studies, although in all cases the delay to treatment was much less than 12 h.

Perhaps participants in our study were too severely asphyxiated and thus had little prospect of benefit from any intervention after birth. We used inclusion criteria modified from those used in cooling trials, which necessitated the presence of the entire main neurological criteria for selection of participants, and that could account for the high rate of severe abnormalities in the aEEG and the high median score for hypoxic ischaemic encephalopathy at trial entry. However, mortality in our cohort was similar to that in previous trials of cooling (although in our trial, death was recorded only until discharge from hospital). The similar death rate to that in the cooling trials despite evidence of worse encephalopathy in our cohort might suggest that early use of routine hypothermia led to better outomes in this trial than in those in which consent had to be obtained before initiation of cooling.

Another possible explanation of our negative results is that the chosen biomarkers are insufficiently sensitive. However, a raised lactate to N-acetyl aspartate ratio was the best predictor of subsequent neurodevelopmental outcome in a meta-analysis^13^ and is a sensitive indicator of subtle effects after birth asphyxia.^13^, ^30^ Furthermore, changes in FA have been used to identify treatment effects in small groups of infants with asphyxia; these changes correlated closely with subsequent outcome.^11^, ^14^ The significant association in our study between these markers and early neurological assessment provides further support for use of these biomarkers. The similar rates of abnormalities in both groups on visual assessment of the MRIs were also consistent with the main findings of the study.

Magnetic resonance biomarkers have great potential for use in the early development of neuroprotectants before undertaking large trials with clinical outcomes. A major challenge is validation of the markers across several magnetic resonance scanners to enable multisite studies. Using a standardised magnetic resonance scanning protocol, we acquired primary outcome data from 85% of participants, showing that cerebral magnetic resonance biomarkers such as lactate to N-acetyl aspartate ratio and FA are useful for rapid, preliminary assessment of potential neuroprotectants and planning of larger definitive trials.

## Figures and Tables

**Figure:**
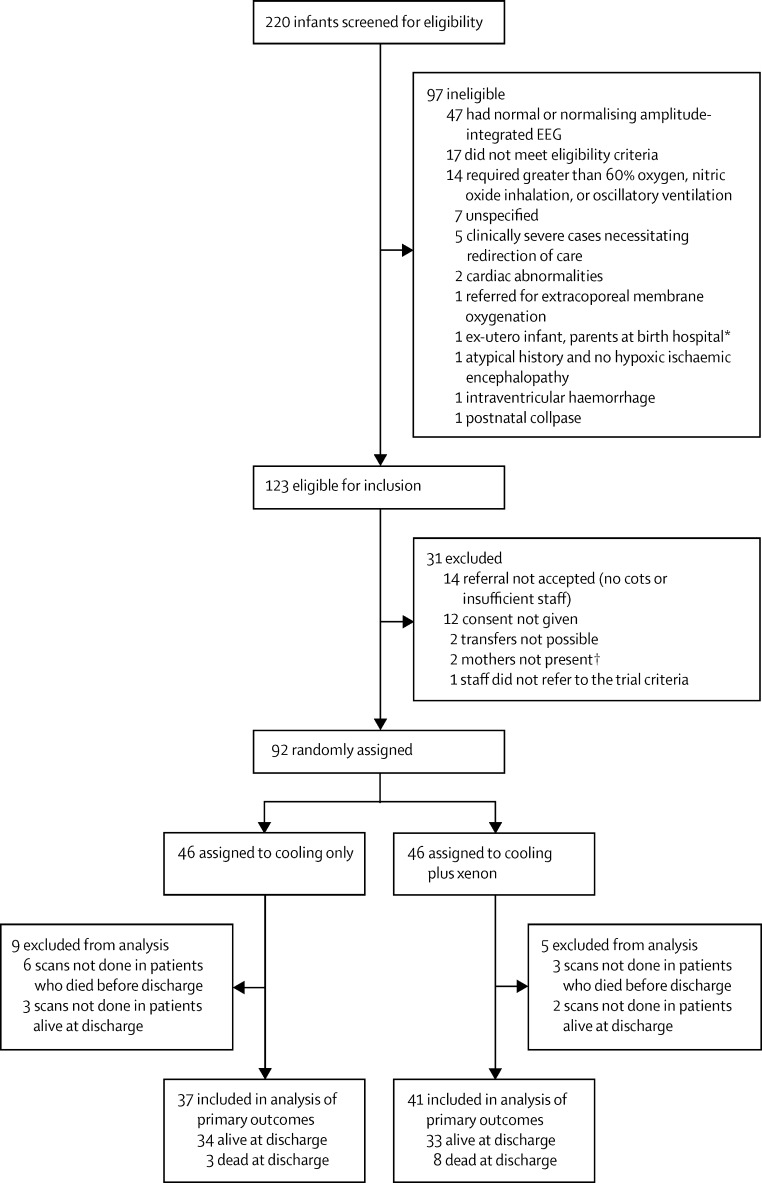
Trial profile *Could not give consent. †When parents were unmarried, only the mother of the infant could provide consent.

**Table 1:** Baseline clinical characteristics in the intention-to-treat population

		**Cooling only (n=46)**	**Cooling plus xenon (n=46)**
Treatment hospital (n)			
	University College London	15	15
	St Thomas′	17	17
	Queen Charlotte and Chelsea	14	13
	Liverpool Women's	0	1
Birth in treatment centre		15 (33%)	16 (35%)
Male sex		21 (46%)	26 (57%)
Birthweight (g), mean (SD)		3213 (448)	3392 (685)
Gestation at delivery (weeks), mean (SD)		39·8 (1·3)	39·8 (1·7)
Apgar at 10 min, median (IQR)		5 (4 to 7)	5 (3 to 6)
Median cord or first blood pH (IQR)		6·9 (6·7 to 7·0)	6·9 (6·8 to 7·1)
Mean cord or first blood pH (SD)		6·9 (0·2)	6·9 (0·2)
Base excess (mmol/L), median (IQR)		–19·7 (–23·7 to −14·0)	–17·7 (–22 to −13·5)
Thompson hypoxic ischaemic encephalopathy score* at trial entry			
	0–10	2 (4%)	5 (11%)
	11–14	30 (65%)	21 (46%)
	15–22	14 (30%)	20 (43%)
	Median (IQR)	14 (12 to 15)	14 (12 to 16)
Abnormality on amplitude-integrated EEG			
	Moderate	7 (15%)	6 (13%)
	Severe	39 (85%)	40 (87%)
Age cooling commenced, n/N (%)			
	<4 h	41/44 (93%)	41/44 (93%)
	4–6 h	3/44 (7%)	3/44 (7%)
	Median (IQR)	0·3 (0·0 to 0·8)	0·2 (0·0 to 1·5)
Head circumference at admission to neonatal unit (cm), mean (SD)		34·4 (1·5)	34·5 (1·8)

Data are n (%), unless otherwise indicated.

* Score ranges from 0 to 22, with higher scores corresponding to more severe encephalopathy.

**Table 2:** Analysis of primary outcomes

		**Cooling only**	**Cooling plus xenon**	**Geometric mean ratio (95% CI)**	**Mean difference (95%CI)**
**Infants with MRI scans**					
Lactate to N-acetyl aspartate ratio				1·09 (0·90 to 1·32)	..
	n	37	41		
	Arithmetic mean (SD)	0·47 (0·94)	0·68 (1·12)		
	Coefficient of variation*	2·19	1·68		
	Geometric mean	0·34	0·47		
Fractional anisotropy				..	–0·01 (–0·03 to 0·02)
	n	35	38		
	Mean (SD)	0·41 (0·01)	0·40 (0·01)		
**Infants with MRI scans surviving to discharge**					
Lactate to N-acetyl aspartate ratio				0·98 (0·85 to 1·12)	..
	n	34	33		
	Arithmetic mean (SD)	0·32 (0·42)	0·34 [0·77]		
	Coefficient of variation*	1·41	1·30		
	Geometric mean	0·28	0·25		
Fractional anisotropy				..	–0·01 (–0·01 to 0·01)
	n	33	30		
	Mean (SD)	0·40 (0·05)	0·40 (0·05)		

Geometric mean ratios were calculated after log (x + 1) transformation. Fractional anisotropy data were extracted from a mask of the posterior limb of the internal capsule via tract-based spatial statistics.

* Coefficient of variation=√(exp(var)–1), where var is the variance on the log scale.

**Table 3:** Analysis of secondary outcomes

		**Cooling only (n=46)**	**Cooling plus xenon (n=46)**	**Relative risk (99% CI)**
Death before discharge		9 (20%)	11 (24%)	1·22 (0·44 to 3·41)
Maximum Thompson hypoxic ischaemic encephalopathy score in first week of life				
	0–10	0 (0%)	1 (2%)	1·22 (0·82 to 1·82)
	11–14	19 (41%)	12 (26%)	
	15–22	27 (59%)	33 (72%)	
	Median (IQR)	16 (13 to 19)	15 (14 to 18)	
Neurological assessment at discharge*				0·66 (0·17 to 2·51)
	Normal or mildly abnormal	29 (78%)	30 (86%)	
	Moderately abnormal	7 (19%)	3 (9%)	
	Very abnormal	1 (3%)	2 (6%)	
Persistent hypotension		29 (63%)	31 (67%)	1·06 (0·72 to 1·58)
Cardiac arrhythmia (heart rate <80 beats per min)		4 (9%)	2 (4%)	0·50 (0·06 to 4·36)
Thrombocytopenia (platelet count <150 × 10^9^ per L)		20 (43%)	18 (39%)	0·90 (0·55 to 1·47)
Prolonged blood coagulation time (activated partial thromboplastin time >41 s or international normalised ratio >3)		32 (70%)	36 (78%)	1·13 (0·82 to 1·55)
Major venous thrombosis		1 (2%)	0 (0%)	..
Anuria or urine output <0·5 mL/kg/h for >24 h, n/N (%)		3/20 (15%)	6/23 (26%)	2·00 (0·38 to 10·5)
Culture-proven late-onset sepsis		0 (0%)	2 (4%)	..
Necrotising enterocolitis		0 (0%)	0 (0%)	..
Pneumonia		1 (2%)	1 (2%)	1·00 (0·03 to 36·71)
Pulmonary air leak		0 (0%)	3 (7%)	..
Pulmonary haemorrhage		3 (7%)	1 (2%)	0·33 (0·02 to 6·21)
Persistent pulmonary hypertension		3 (7%)	3 (7%)	1·00 (0·13 to 7·64)
Intracranial haemorrhage		3 (7%)	4 (9%)	1·33 (0·20 to 8·85)
Seizures		36 (78%)	36 (78%)	1·00 (0·75 to 1·33)
Median age (IQR) full oral feeding achieved (days)†		9 (7 to 11)	9 (7 to 12)	..
Did not achieve full oral feeding by discharge†		6 (17%)	4 (12%)	0·73 (0·16 to 3·40)
Median hospital stay (IQR) to discharge* (days)		14 (10 to 17)	12 (9 to 22)	–1 (–5 to 4)‡

Data are n (%) unless otherwise specified. Hypotension was defined as a mean blood pressure of less than 40 mmHg. Seizures included both clinical and subclinical events, and were identified by amplitude-integrated EEG.

* Calculated only in infants alive at discharge.

† Data available for 36 infants in the cooling group and 33 in the xenon group. Median (IQR) based only on those who achieved full oral feeding by discharge.

‡ These data are median difference (95% CI).

**Table 4:** Visual analysis of MRI by score (secondary outcome)

	**Cooling only (n=39)**	**Cooling plus xenon (n=44)**	**Relative risk (99% CI)**	**Mean difference in scores (99% CI)**
**Posterior limb of internal capsule**				
Score 0	18	21		0·07 (–0·44 to 0·57)
Score 1	11	8	0·97 (0·57 to 1·65)	0·07 (–0·44 to 0·57)
Score 2	10	15	0·97 (0·57 to 1·65)	0·07 (–0·44 to 0·57)
**Basal ganglia and thalamus**				
Score 0	6	14		–0·05 (–0·71 to 0·60)
Score 1	9	3		–0·05 (–0·71 to 0·60)
Score 2	16	13	1·00 (0·64 to 1·56)	–0·05 (–0·71 to 0·60)
Score 3	8	14	1·00 (0·64 to 1·56)	–0·05 (–0·71 to 0·60)
**White matter**				
Score 0	16	14		0·33 (–0·35 to 1·00)
Score 1	7	8		0·33 (–0·35 to 1·00)
Score 2	11	10	1·22 (0·65 to 2·29)	0·33 (–0·35 to 1·00)
Score 3	5	12	1·22 (0·65 to 2·29)	0·33 (–0·35 to 1·00)
**Cortex**				
Score 0	29	30		0·33 (–0·33 to 0·99)
Score 1	4	2		0·33 (–0·33 to 0·99)
Score 2	3	2	1·77 (0·56 to 5·64)	0·33 (–0·33 to 0·99)
Score 3	3	10	1·77 (0·56 to 5·64)	0·33 (–0·33 to 0·99)

Data are n. Relative risk is calculated for the moderate and severe changes groups combined, so only one relative risk and 99% CI is listed for each brain site. For the posterior limb of the internal capsule scores, 0=normal, 1=equivocal (reduced or asymmetrical signal intensity), and 2=loss (reversed or abnormal signal intensity bilaterally on T1-weighted or T2-weighted sequences, or both). For basal ganglia and thalamic scores, 0=normal, 1=mild (focal abnormal signal intensity), 2=moderate (multifocal abnormal signal intensity), and 3=severe (widespread abnormal signal intensity). For white matter scores, 0=normal, 1=mild (exaggerated long T1 and long T2 in periventricular white matter only), 2=moderate (long T1 and long T2 extending out to subcortical white matter or focal punctate lesions or focal area of infarction, or any combination thereof), and 3=severe (widespread abnormalities including overt infarction, haemorrhage, and long T1 and long T2). Cortical involvement was scored as the presence of abnormal signal intensity, usually decreased T1 or cortical highlighting (ie, increased signal intensity in the cortex). For cortical scores, 0=normal, 1=mild (one or two sites involved), 2=moderate (three sites involved), and 3=severe (more than three sites involved). The sites included the central sulcus, interhemispheric fissure, and the insula. All the scans were assessed and graded by NT, who was masked to intervention.
