# Supplementary material for: Moderate hypothermia within 6 h of birth plus inhaled xenon versus moderate hypothermia alone after birth asphyxia (TOBY-Xe): a proof-of-concept, open-label, randomised controlled trial
Source: Lancet Neurol. 2016 Feb;15(2):145–53. doi: 10.1016/S1474-4422(15)00347-6 (PMC4710577; doi:10.1016/S1474-4422(15)00347-6)
Supplement: Supplementary appendix [file mmc1.pdf]

THE LANCET Neurology

Supplementary webappendix

This webappendix formed part of the original submission and has been peer reviewed. We post it as supplied by the authors.

Supplement to: Azzopardi D, Robertson NJ, Bainbridge A, et al. Moderate hypothermia within 6 h of birth plus inhaled xenon versus moderate hypothermia alone after birth asphyxia (TOBY-Xe): a proof-of-concept, open-label, randomised controlled trial. *Lancet Neurol* 2015; published online Dec 18. http://dx.doi.org/10.1016/S1474-4422(15)00347-6.

Appendix

Scanning procedures

MRI examination was structured to acquire the anatomical/clinical data first, then the quantitative measures and finally additional scans to provide motion tolerant images to provide diagnostic information in those subjects who were not still during the examination.

A specific TobyXe exam card was used. This exam card uses Philips smartExam technology to aid the planning of the various sequences, and determines the brain position and evidence of any change in position during scanning. A quality assessment was carried out after each acquisition looking for signs of patient motion, signal inhomogeneity, and poor signal to noise ratio. The acquisition was repeated if necessary.

Sequences

The sequence parameters and orientation are shown in the table:

MR scanning parameters

Sequence name	Sequence type	TR (ms[a])	TI (ms)	TE (ms)	Acquired Resolution (mm^3)	Orientation
T2 weighted	Multi-slice FSE	15715	-	160	1.15x1.18x2.0	Transverse
T1 weighted	MP-RAGE	17	1465	4.6	0.82x0.97x1.0	Sagittal
DT-MRI*	Spin echo EPI	7857	-	49	2.0x2.03x2.0	Transverse
MRS	PRESS	2288	-	288	15x15x15	Acquired in left thalamus
Motion tolerant T2	Single Shot FSE	27744	-	160	0.98x0.99x2.00 (50% slice overlap)	Transverse
Motion tolerant T1	Single Shot FSE	22000	400	6.1	0.98x0.98x2.00	Transverse

* The b-value for DT-MRI was 750 s/mm^2
[a] ms = milliseconds

MR spectroscopy

Proton (1H) MRS was obtained from a 15 x 15 x 15 mm voxel centred on the left thalamus using a Point resolved spectroscopy (PRESS) acquisition (repetition time = 2288ms, echo time = 288ms, acquisition bandwidth = 4kHz, 2048 datapoints). Sixteen sub-spectra were acquired as a dynamic series, each with 8 signal averages. Pre-processing of the sub-spectra was performed using the Jmrui software.[1] This pre-processing consisted of manual phasing and frequency correction (to ensure that the prominent spectral peaks were aligned in each sub-spectrum) before summing the sub-spectra to yield the final spectrum. This final spectrum was then fitted using LCModel.[2] The LCModel basis set included basis functions for N-acetyl-aspartate (Naa), N-acetyl-aspartyl-glutamate (NaaG) and lactate (Lac). The ratio of total Naa (Naa + Naag) / Lac was then calculated from the LCModel output.

Tract Based Spatial Statistics (TBSS) Analysis of MRI data

Pre-processing

DTI analysis was performed using FMRIB's Diffusion Toolbox (FDT v2.0) and Tract-Based Spatial Statistics (TBSS v1.2) as implemented in FMRIB's Software Library (FSL v4.1; www.fmrib.ox.ac.uk/fsl.[3] Each infant's diffusion weighted images was registered to their b = 0 image

and corrected for differences in spatial distortion due to eddy currents. Images were brain extracted using Brain Extraction Tool (BET v2.1) and diffusion tensors calculated voxelwise, using a simple least squares fit of the tensor model to the diffusion data. From this, the tensor eigenvalues, describing the diffusion strength in the primary, secondary and tertiary diffusion directions, and fractional anisotropy maps were calculated.

Image registration

Prior to warping, individual fractional anisotropy maps were aligned to each other with an initial 12 degrees-of-freedom (DOF) affine registration using FMRIB's Linear Registration Tool (FLIRT v5.5; default parameters). To achieve accurate spatial alignment, fractional anisotropy maps were then registered using FMRIB's Non-Linear Registration Tool (FNIRT v1.0; parameters as defined in FA_2_FMRIB58_1mm configuration file).

To identify an appropriate individual FA map to act as a study-specific target, every subject's FA map was registered to the map of every other subject. Each warp field (final resolution: $8.75 \times 8.75 \times 10$ mm^3) was summarised by a score representing the mean square spline coefficients of displacement across 3 dimensions and the target image chosen as the one with the minimum mean displacement score from all other subjects in the group, thus deemed to be the most 'typical' image of the group.

Additional linear and non-linear registrations steps were added to reduce the number of failed registrations obtained using the standard TBSS protocol and improve global alignment.[4]

Voxelwise analysis

Individual FA maps were aligned into the target space and up-sampled to $1 \times 1 \times 1$ mm^3 voxel size using the previously estimated warps. An average FA map was created and thinned by perpendicular non-maximum suppression to generate a mean FA skeleton to represent the centre of all white matter tracts common to the group. This skeleton was thresholded at FA > 0.15 and manually cleaned to include the major white matter pathways but exclude cerebral sinuses and cerebrospinal fluid art factually represented in the skeleton. Individual FA, axial diffusivity – the magnitude of the principal vector of the diffusion tensor, $\lambda 1$ – and radial diffusivity – the mean magnitude of the transverse vectors, $(\lambda 2 + \lambda 3) / 2$ – data were then projected onto this skeleton prior to statistical analysis.

Voxelwise cross-subject statistical analysis was performed to compare the 2 groups with Randomise (v2.1) using univariate linear modelling in the form of a general linear model. All images were subject to family-wise error (FWE) correction for multiple comparisons following threshold-free cluster enhancement in image space.[5]

[1]: Naressi, A., Couturier, C., Devos, J.M., Janssen, M., Mangeat, C., de Beer, R., Graveron-Demilly, D. Java-based graphical user interface for the MRUI quantitation package. MAGMA, 2001; 12(2-3): 141-52.
[2]: Provencher, S.W. Automatic quantitation of localized in vivo 1H spectra with LCModel. NMR Biomed, 2001;14: 260-264.
[3] Smith SM, Jenkinson M, Woolrich MW, Beckman CF, Behrens TEJ, Johansen-Berg H, et al. Advances in functional and structural MR image analysis and implementation as FSL. Neuroimage. 2004;23(S1):208-19.
[4] Ball, G, Counsell, SJ., Anjari M; et al An optimised tract-based spatial statistics protocol for neoenates: Applications to prematurity and chronic lung disease 2010; 53(1): 94-102.
[5] Smith SM, Nichols TE. Threshold-free cluster enhancement: addressing problems of smoothing threshold dependence and localisation in cluster inference. Neuroimage. 2009;44(1):83-98.
